# Test-retest reliability of time-varying patterns of brain activity across single band and multiband resting-state functional magnetic resonance imaging in healthy older adults

**DOI:** 10.3389/fnhum.2022.980280

**Published:** 2022-11-10

**Authors:** Marie-Stephanie Cahart, Flavio Dell’Acqua, Vincent Giampietro, Joana Cabral, Maarten Timmers, Johannes Streffer, Steven Einstein, Fernando Zelaya, Steven C. R. Williams, Owen O’Daly

**Affiliations:** ^1^Department of Neuroimaging, Institute of Psychiatry, Psychology and Neuroscience, King’s College London, London, United Kingdom; ^2^NatBrainLab, Department of Forensic and Neurodevelopmental Sciences, Institute of Psychiatry, Psychology and Neuroscience, King’s College London, London, United Kingdom; ^3^Life and Health Sciences Research Institute, University of Minho, Braga, Portugal; ^4^Janssen Research and Development, A Division of Janssen Pharmaceutica NV, Beerse, Belgium; ^5^AC Immune SA, Lausanne, Switzerland; ^6^Reference Center for Biological Markers of Dementia (BIODEM), Institute Born-Bunge, University of Antwerp, Antwerp, Belgium; ^7^UCB Biopharma SPRL, Brussels, Belgium

**Keywords:** phase synchronization, brain dynamics, functional connectivity, resting-state, reliability, single band, multiband (MB)

## Abstract

Leading Eigenvector Dynamics Analysis (LEiDA) is an analytic approach that characterizes brain activity recorded with functional Magnetic Resonance Imaging (fMRI) as a succession of discrete phase-locking patterns, or states, that consistently recur over time across all participants. LEiDA allows for the extraction of three state-related measures which have previously been key to gaining a better understanding of brain dynamics in both healthy and clinical populations: the probability of occurrence of a given state, its lifetime and the probability of switching from one state to another. The degree to which test-retest reliability of the LEiDA measures may be affected by increasing MRI multiband (MB) factors in comparison with single band sequences is yet to be established. In this study, 24 healthy older adults were scanned over three sessions, on weeks 0, 1, and 4. On each visit, they underwent a conventional single band resting-state fMRI (rs-fMRI) scan and three different MB rs-fMRI scans, with MB factors of 4, with and without in-plane acceleration, and 6 without in-plane acceleration. We found test-retest reliability scores to be significantly higher with MB factor 4 with and without in-plane acceleration for most cortical networks. These findings will inform the choice of acquisition parameters for future studies and clinical trials.

## Introduction

Traditionally, resting-state functional MRI (rs-fMRI) studies have assumed that the functional connectivity (FC) between different regions of the brain is constant over time. However, over the last decades FC studies have begun investigating variations in FC in the order of seconds and have revealed that brain activity does fluctuate over time (Hutchison et al., 2013; Preti et al., 2017) and that brain regions can synchronize in activity even in the absence of any specific task or stimulus (Biswal et al., 1995; Chang and Glover, 2010). Since these early discoveries, a significant number of studies have emerged, aiming to specifically investigate this so-called dynamic FC (Liegeois et al., 2017; Yue et al., 2018; Lurie et al., 2020). Rs-fMRI research has demonstrated that there exist meaningful functional networks, also called resting-state networks (RSNs), that reflect spontaneous patterns of coordinated activity at rest. Methods focusing on the time-varying activation of RSNs have been shown to be sensitive to disruption of healthy brain functioning (Derome et al., 2018; Figueroa et al., 2019; Li et al., 2019; Alonso-Martínez et al., 2020).

One common approach to studying dynamic FC is the sliding-window method (Hutchison et al., 2013; Shakil et al., 2016), which involves segmenting the resting-state time series from different brain regions or voxels into shorter subsets of consecutive volumes (windows), with connectivity estimated for each of the truncated timeseries. This technique has shed light on some of the fundamental properties of brain network functions, such as revealing unique profiles of dynamic connectivity for different subdivisions of the insula (Nomi et al., 2016) and network dysfunction in several psychiatric disorders. Indeed, atypical temporal variability of the FC of the amygdala has been observed in schizophrenia (Yue et al., 2018) and altered medial prefrontal cortex FC dynamics have been identified in major depression (Kaiser et al., 2016). However, limitations of this method include the initial choice of the window length and the length of the steps by which the window moves, which may affect statistical outcomes such as the sensitivity and specificity of the results (Hindriks et al., 2016; Preti et al., 2017).

To address these issues, Cabral et al. (2017) introduced a new method for studying dynamic FC called Leading Eigenvector Dynamics Analysis (LEiDA). This approach aims to study brain dynamics as a succession of discrete phase-locking states in fMRI signals that recur over time across all subjects. More specifically, LEiDA explores time-varying FC by examining the instantaneous phase relationship between brain regions and identifying specific epochs when a particular FC pattern, or state, starts dominating the variance of the dynamic FC at the brain level. This approach limits issues related to high dimensionality of the data by calculating, at each timepoint, the largest magnitude eigenvector of phase alignment across all regions of interest (ROIs) and only examining the relative phase of each ROI with respect to the leading eigenvector (Cabral et al., 2017). This leads to the detection of meaningful BOLD phase-locking patterns, or states, that have previously been shown to closely overlap with functional subsystems described in the literature (Yeo et al., 2011; Lord et al., 2019).

The LEiDA approach has been used in the past to gain a better understanding of brain dynamics across a range of healthy and clinical populations. It was initially applied to a neuropsychology study, which demonstrated a close relationship between the switching profile of resting-state functional patterns in healthy older adults and their performance on neuropsychological tests (Cabral et al., 2017). The method has also contributed to providing a novel neurobiological profile of trait self-reflectiveness (Larabi et al., 2020), hedonic processing (Stark et al., 2019), and vulnerability to major depressive disorders (Figueroa et al., 2019). Evidence increasingly points toward the study of metastable phase coupling and uncoupling as a solid and robust signature of typical and atypical brain function that warrants further investigation (Deco et al., 2019; Hancock et al., 2022).

It is worth noting that LEiDA allows for the extraction of three measures: the probability of occurrence of a given state, its lifetime and the probability of switching from one state to another. Using an open-source fMRI dataset from 99 healthy participants from the Human Connectome Project, Vohryzek et al. (2020) explored the test-retest reliability of all three measures over time using the Intraclass Correlation Coefficient (ICC; Shrout and Fleiss, 1979). The ICC scores were derived from two resting-state fMRI scans with a multiband (MB) factor of 8 and a TR of 0.72 s, acquired on the same day within the same session and differing only in the oblique axial acquisition phase encoding (left to right in one run and right to left in the other run). ICC scores for the probability of occurrence and lifetime measures showed “fair” to “moderate” reliability scores overall, while the switching probability matrix exhibited “poor” to “substantial” reliability scores. Achieving satisfactory test-retest reliability is crucial when considering LEiDA’s possible clinical applications. Indeed, reliability of results ensures effective and trustworthy contribution to scientific and clinical knowledge, including the ability to replicate and integrate data into larger investigations (Bennett and Miller, 2010).

It is important to note that MB acquisitions were first introduced by Larkman et al. (2001) in 2001 and have since become increasingly used due to their ability to increase the temporal resolution of fMRI scans and therefore accelerate scanning time. MB sequences consist of simultaneously exciting and acquiring multiple slices of the brain by using a MB radiofrequency pulse, instead of exciting only one slice of the brain at a time as observed with conventional single band acquisitions. They are typically acquired using a high number of channels in the head coil, such as a 32-channel head coil with a higher number of rows of coils along the *z*-axis. The number of slices simultaneously excited is referred to as the MB factor and, for a given period of acquisition time, the number of volumes collected increases by the MB factor. For this reason, MB protocols can significantly improve temporal resolution [i.e., decreased the repetition time (TR) needed] for whole-brain imaging. However, they may also lead to reduced temporal signal-to-noise ratio because of signal dropout (Chen et al., 2015). To mitigate issues associated with susceptibility-related distortions as well as signal dropouts, in-plane acceleration has also been used in recent studies (Todd et al., 2016). In-plane acceleration typically consists in combining data in image space as it is typically performed with “Sensitivity Encoding” or “SENSE” acceleration or combining data in k-space as it is commonly achieved with “Generalized Auto-calibrating Partial Parallel Acquisition” (GRAPPA). Previous studies have suggested that a total acceleration of 4 (i.e., MB factor 2 with in-plane acceleration of 2) allows for an optimal detection of common RSNs and is associated with a negligible decrease in signal to noise ratio in comparison with a total acceleration of 2, 6, and 8 (Preibisch et al., 2015). Additionally, recent research has also demonstrated the key role of MB sequences in improving the test-retest reliability of FC measures for cortical structures in the context of commonly used static, as opposed to dynamic, FC measures (Cahart et al., 2022). More specifically, MB 4 with no in-plane acceleration was the MB sequence that yielded the highest reliability scores for cortical structures, while MB 4 with an in-plane acceleration of 2 displayed the lowest values. In contrast, single band acquisition was recommended when the experimental focus was specifically on subcortical regions. However, it is not yet known how different MB factors combined with different levels of in-plane acceleration influence test-retest reliability of metrics of dynamic FC.

Additionally, recent research suggests that the number of scanning sessions, as well as the time frame within which the scans occur, are important experimental components that can significantly influence test-retest reliability (Bennett and Miller, 2010). Indeed, within-subject factors such as attention, arousal, physiological, and cognitive changes are known to have an impact on test-retest reliability across sessions. In particular, it has been suggested that the greater the amount of time between the initial scan and the subsequent retest scan, the larger these changes are likely to be, thus potentially lowering ICC scores (Bennett and Miller, 2010).

The present study aimed to address gaps in the literature by investigating the test-reliability of all three LEiDA measures over three runs acquired at the same time of day on weeks 0, 1, and 4, across both single band and MB modalities with and without in-plane acceleration.

Based on the previous research described above, we hypothesized that (1) ICC scores would be significantly higher for MB compared to single band and (2) MB ICC scores would be highest with a total acceleration of 4 (i.e., MB 4 with no in-plane acceleration) and lowest with a total acceleration of 8 (i.e., MB 4 with an in-plane acceleration of 2) across all three LEiDA measures.

## Materials and methods

### Participants

Twenty-four healthy older adults (*M* = 16, *F* = 9) aged between 52 and 73 took part in the study after providing written informed consent (ethics number HR-17/18-5720; King’s College London Research Ethics Committee). All participants were right-handed, with no history of psychiatric disorder or neurological disease and were not taking any psychoactive treatments such as antidepressants.

### Procedure

Each participant attended three scanning sessions at the Centre for Neuroimaging Sciences (Institute of Psychiatry, Psychology and Neuroscience; King’s College London), on weeks 0, 1, and 4, at the same time of day, ± 1 h, ± 1 day.

### Magnetic resonance imaging data acquisition

All participants were scanned in the same 3T MR scanner (Discovery MR750, General Electric, Milwaukee, WI, USA). On each visit, they underwent an anatomical T1-weighted MRI with the following parameters: repetition time = 8.23 ms; echo time = 3.25 ms; flip angle = 12°; field of view = 230 mm^2^; matrix size = 256 × 256; slice thickness = 0.9 mm; 1 mm isotropic resolution. Participants also underwent four resting-state FC sequences: (1) standard Echo-Planar Imaging, in-plane acceleration 2 (SB, ASSET = 2); (2) MB 4, no in-plane acceleration (MB = 4, ARC = 1); (3) MB 4, in-plane acceleration 2 (MB = 4, ARC = 2); and (4) MB 6, no in-plane acceleration (MB = 6, ARC = 1). In this paper, ASSET (“Array Coil Spatial Sensitivity Encoding”) refers to the methodology we employed for the single band sequence. It is the General Electric commercial name for SENSE acceleration. It consists in parallel imaging with in-plane acceleration, combining data in image space as it is commonly done when using SENSE acceleration. For MB sequences, because ASSET is not compatible with MB, we employed ARC (“Auto-calibrating Reconstruction for Cartesian Imaging”), which refers to parallel imaging with data combination in k-space as performed by GRAPPA. ARC is the General Electric commercial name for GRAPPA.

The parameters for each of the rs-fMRI runs are presented in Table 1. The order of the four sequences was counterbalanced across imaging sessions and subjects. Each run was 8-min long. For this study, we used the Nova 32-channel head coil.

**TABLE 1:** Parameters of resting-state fMRI sequences.

	TR	TE	FA	FoV	Matrix size	Time points
SB-ASSET2	2,000 ms	30 ms	82°	211 × 211 × 126	64 × 64	240
MB4-ARC1	750 ms	30 ms	63°	211 × 211 × 145	64 × 64	644
MB4-ARC2	750 ms	30 ms	63°	211 × 211 × 145	64 × 64	645
MB6-ARC1	550 ms	30 ms	57°	211 × 211 × 138	64 × 64	873

TR, repetition time; TE, echo time; FA, flip angle; FoV, field of view. Each run was 8-min long and carried out using a 32-channel head coil from Nova.

During the acquisition of the resting-state sequences, the participants were asked to look at a white cross on a dark screen in a wakeful resting state and were provided with headphones and earplugs in order to reduce the acoustic noise generated by the scanner.

### Magnetic resonance imaging data pre-processing

Statistical Parametric Mapping (SPM12; Wellcome Trust Centre for Neuroimaging, London, UK) and the CONN toolbox Version 18b (Whitfield-Gabrieli and Nieto-Castanon, 2012) were used to pre-process the data. Pre-processing of the functional data included realignment (motion correction), registration to structural images, spatial normalization into the Montreal Neurological Institute (MNI) standardized space and smoothing with a Gaussian filter of 5.0 mm spatial full width at half maximum value. As slice timing effects are considerably larger for single band sequences compared to MB, slice-timing correction was performed only on the standard echo-planar imaging sequence. The artifact rejection tool (ART), implemented in CONN,^1^ was used to identify outliers based on subject movement and changes in the fMRI signal. Volumes with a framewise displacement (FD) above 0.9 mm or global BOLD changes above 5 standard deviations were flagged as potential outliers. One covariate per outlier volume was entered in the denoising regression step so as to limit the influence of those scans on the analyses. In particular, FD is computed by the ART toolbox and is calculated by considering a 140 × 180 × 115 bounding box around the brain for each timepoint, and estimating the maximum brain displacement across six control points placed at the center of each face of the bounding box (Nieto-Castanon, 2020). Furthermore, the anatomical CompCor method (aCompCor; component-based noise correction method; Behzadi et al., 2007) was used to estimate and regress out physiological and other irrelevant sources of noise. More specifically, a binary mask with values higher than 50% in white matter and CSF posterior probability maps was applied in order to define potential confounding effects from the observed BOLD signal within each of those two areas. Within each area, a principal component decomposition was carried out on the subspace orthogonal to the mean BOLD signal and to all other known potential confounding factors. Five potential noise components (Chai et al., 2012) were estimated: the first one was calculated as the average BOLD signal, and the other four were computed as the first four components in a Principal Component analysis of the covariance within that subspace. A conventional bandpass filter over a low-frequency window of interest (0.008–0.09) was then applied to the resting-state time series for the four rs-fMRI modalities.

In order to check that aCompCor had successfully reduced motion artifacts, given that head motion may distort rs-fMRI data, the distribution of the correlations between FD and percent signal change [as measured by the Derivative of root mean square VARiance over voxelS (DVARS)] for each participant was plotted for run 1 for each rs-fMRI modality (Hallquist et al., 2013; Muschelli et al., 2014) using MATLAB R2020a (MathWorks, Natick, MA, USA). The FD timeseries were extracted from the CONN toolbox for each rs-fMRI modality and each subject. The DVARS timeseries for each rs-fMRI modality and each subject were calculated before and after denoising using a script provided by Afyouni and Nichols (2018). In order to further explore how much variability in DVARS is explained by FD, the Coefficients of Determination were also calculated for each rs-fMRI modality and each subject, using the mdl.Rsquared MATLAB command. Finally, in line with previous work by Muschelli et al. (2014), Wilcoxon’s signed-rank tests were performed to formally assess differences in group medians with regards to the distribution of the correlations FD-DVARS and the Coefficients of Determination. We used false discovery rate (FDR) correction (Benjamini and Hochberg, 1995) to adjust for all four rs-fMRI modalities.

### Dynamic analysis

The analysis of dynamic connectivity was carried out in MATLAB R2020a (MathWorks, Natick, MA, USA) using LEiDA scripts adapted from Cabral et al. (2017). The scripts can be found here: https://osf.io/xy4gm/.

*N* = 105 ROIs were extracted from the CONN toolbox (Whitfield-Gabrieli and Nieto-Castanon, 2012) and were anatomically defined based on the Harvard-Oxford cortical atlas. The BOLD signals were averaged over all voxels belonging to each ROI. The cerebellar ROIs were not included in line with previous work (Cabral et al., 2017) due to the absence of cerebellar networks in the Yeo parcellation used as part of our analyses (Yeo et al., 2011).

In order to capture recurrent phase-locking patterns, we first estimated the phase of the fMRI BOLD signals over time for each brain region (*N* = 105) for each of the four rs-fMRI modalities using the Hilbert transform. More precisely, the timeseries were first demeaned and then transformed into an analytic signal which, unlike the real, measured, signal, has an associated phase. The Hilbert transform expresses the signal as the product of the time-varying amplitude A(t) and the cosine of a time-varying phase angle θ(t) as follows:


x⁢(t)=A⁢(t)⁢cos⁡θ⁢(t)

Indeed, it computes an imaginary component of the real signal assuming that the signal oscillates over time: the real signal is represented by the cosine of the phase angle, while the imaginary signal is represented by the sine. Since the signal is analyzed in the complex domain, the analytic signal results from adding together the real and imaginary components:


$$A^{*}\,\cos\left(\theta \right)+l\,i^{*}\,A^{*}\,\sin\left(\theta \right)$$

**FIGURE 1:**
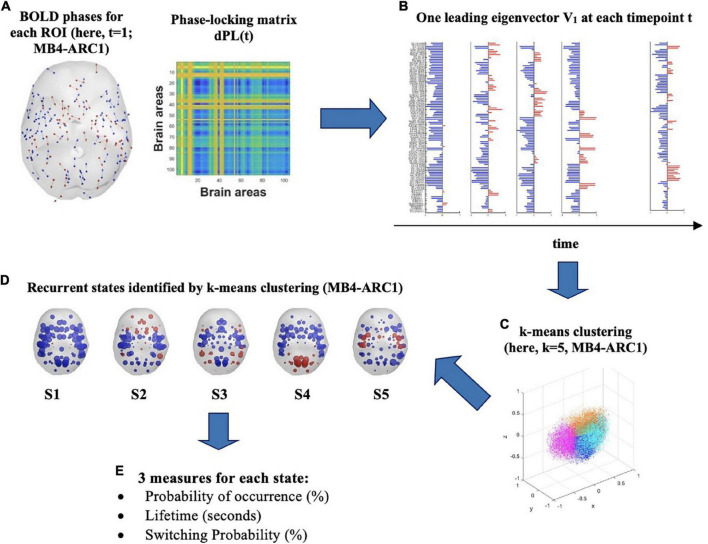
Identification of recurrent BOLD phase-locking patterns (or states) in fMRI signals. **(A)** (left) Instantaneous BOLD phases represented in cortical space for each of the 105 ROIs. Each arrow represents the phase orientation of each ROI at timepoint t and is centered around the center of gravity of each ROI. Here, first volume (*t* = 1) for MB4-ARC1. (right) The 105 × 105 dynamic phase-locking matrix dPL(t) captures the degree of synchrony between pairs of ROIs at time t. Red entries reflect full synchrony between pairs of ROIs (phase difference of 0°°) while blue entries indicate a phase difference of 180°. **(B)** At each timepoint t, the leading eigenvector V_1_(t) is a 105 × 1 vector which captures the dominant pattern of the dPL matrix, or main orientation of all BOLD phases, at time t. Each element in the bar plot represents the contribution of each ROI to V_1_(t) and is colored depending on its relative direction with respect to V_1_(t). Red means that the contribution is positive, while blue means it is negative. **(C)** K-means clustering divides the sample of all leading eigenvectors V(t) into a reduced number of k clusters, or states. Here, the Dunn score identified *k* = 5 as the optimal number of states to best explain the MB4-ARC1 data. **(D)** Each sphere represents the center of gravity of a ROI, and each color represents the direction of the projection of the ROI’s phase onto the leading eigenvector V_1_ of that state. Here, *k* = 5 for MB4-ARC1. **(E)** Three measures are then extracted for each of these states: the probability of occurrence of each state, its lifetime and the probability of switching from one state to another.


Once the analytic signal was computed and the BOLD phases were identified for each ROI, the second step consisted in identifying the degree of synchrony between pairs of brain regions at each timepoint t. First, we computed a symmetric BOLD phase-lockingmatrix [dPL(t)], which shows phase alignment, or degree of BOLD synchrony, between pairs of ROIs across the entire brain for each participant at each timepoint t, as illustrated in Figure 1A. For every pair of ROIs a and b at time t, the dPL was calculated as the cosine of the difference in phases of two regions, as follows:

d⁢P⁢L⁢(b,a,t)=cos⁡(θ⁢(b,t)-θ⁢(a,t))

At a given timepoint, two brain regions will have a phase-locking value of 1 if their BOLD signals are in full synchrony, or their phases are fully aligned, with no phase difference in complex plane [i.e., cos(0) = 1]. In contrast, a value of −1 reflects a phase difference of 180°, or anti-phase. Phase-locking measures synchronization by preserving the positive and negative weights in the data and is bound between −1 and 1 (Hancock et al., 2022). An illustration of phase-locking patterns between two signals is provided in Supplementary Figure 1.

To capture the phase-lockingpatterns of the dPL at every timepoint t with reduced dimensionality, the leading eigenvector V_1_(t) was then calculated for each dPL(t). More specifically, for each dPL(t), the leading vector V_1_(t), of dimension Nx1, captures the main orientation of the phases across all 105 ROIs (Figure 1B). LEiDA considers only the eigenvector with the largest magnitude eigenvalue rather than considering all upper triangular elements of the dPL. This approach allows for the dimensionality of the data to be reduced from N(N-1)/2 to N. The NxN dominant connectivity pattern of phase-locking can be retrieved by multiplying the eigenvector with its transpose [V_1_(t)*V_1_*^T^*(t)] (Cabral et al., 2017). The sign (positive or negative) of each element in V_1_(t) represents the direction of the projection of each phase onto V_1_(t) and can thus be used to separate the ROIs into two groups according to their phase relationship and the direction their phase projects onto V_1_(t) (Newman, 2006). Although the relative sign of the elements in the eigenvector is arbitrary, a convention setting most of the elements in V_1_(t) to negative values has previously been established for consistency (Alonso-Martínez et al., 2020; Vohryzek et al., 2020). A positive sign highlights the network of ROIs whose phases project onto the opposite direction from the leading eigenvector. It is worth noting that this group of areas with a positive sign has previously been shown to represent meaningful functional brain networks dominating at a timepoint t, and significantly overlapping with functional networks identified in the literature (Lord et al., 2019; Vohryzek et al., 2020).

For each of the four rs-fMRI modalities, k-means partition was then applied to all leading eigenvectors V_1_(t) in order to divide the phase-space into an optimal number of clusters, or states, k. The k-means algorithm relies on an iterative process to find the solution that minimizes the distance between each observation and the closest cluster centroid. In the present study, k-means partition was used in order to iteratively cluster the leading eigenvectors into *k* = 5 to *k* = 10 clusters, repeating each calculation 1,000 times to ensure stability of the results. The number of states initially chosen for the k-means clustering analysis (i.e., *k* = 5–10) was based on a trade-off between more-fined grained analysis made possible with higher k and the robustness of the state solutions achieved with lower k (Vohryzek et al., 2020). Finally, the Dunn score was calculated in order to identify which optimal number of clusters k best explains the number of states the brain travels across over the entire duration of the scan (Figures 1C,D). The number of clusters that yielded the highest Dunn index was selected, since a higher index reflects more optimal clustering defined by smaller variance within each cluster and higher inter-cluster distance (Dunn, 1973). As such, the Dunn score is calculated as the ratio between the minimal inter-cluster distance and the maximal intra-cluster distance as follows:


$$D\,u\,n\,n_{K}=\min_{1<i,j<K}\left\{\frac{\min_{x\in C_{i},y\in C_{j}}d\,\left(x,y\right)}{\max_{1<k<K}\max_{x,y\in C_{k}}d\,\left(x,y\right)}\right\}$$

where d(x,y) represents the Euclidean distance between the vectors x and y (Salehi et al., 2018).

Following the cluster partition into k states, three measures were then extracted for each cluster or state: the probability of occurrence, which represents the fraction of timepoints in which a state is active during the scan; the lifetime, which refers to the mean number of consecutive timepoints in which a state is active during the scan; and the switching probability, which represents the probability of switching from one state to another, normalized by the probability of the occurrence of the state from which the transitioning is taking place (Figure 1E).

### Comparing clusters across all four resting-state functional magnetic resonance imaging modalities and in relation to reference functional networks

In order to be able to formally compare ICC scores across all four rs-fMRI modalities, given that networks identified for one fMRI sequence may be differently ordered in the k-means output compared to the other three fMRI sequences, we calculated, for each of the 105 ROIs, the proportion of ROIs that shared spatial similarities between pairs of states across all four rs-fMRI modalities using the Pearson’s correlation coefficient. We then compared each state to seven reference functional networks as defined by Yeo et al. (2011), which include the visual, somato-motor, dorsal attention, ventral attention, limbic, fronto-parietal, and DMN networks. In order to do so, each of the seven networks was transformed into a vector with *N* = 105 elements which represented how much each of the *N* = 105 ROIs contributed to each of the seven Yeo networks. We then calculated the Pearson’s correlation coefficients between each Yeo network and each state across all four resting-state modalities. Significance was set at *p* < 0.01/k, k being the optimal number of states identified by the Dunn score for each resting-state modality. Only networks where a significant correlation (*p* < 0.01/k) was observed across all four MRI sequences were taken forward to the reliability analysis.

### Intraclass correlation coefficient analysis

We then calculated the ICC score (Landis and Koch, 1977) of each of the extracted measures (i.e., probability of occurrence, lifetime, and switching probability) for each state across all sessions for each rs-fMRI modalities (i.e., SB-ASSET2, MB4-ARC1, MB4-ARC2, and MB6-ARC1). The ICC scores were calculated using a Matlab code developed by Salarian (2016). The ICC score describes the proportion of within-subject variability vs. between-subject variability as follows, where MSE_*b*_ and MSE_*w*_ are the between-subject and within-subject mean squared errors, respectively (Charman et al., 2017):


$$I\,C\,C=\frac{M\,S\,E\,b-M\,S\,E\,w}{M\,S\,E\,b+M\,S\,E\,w}$$

$$M\,S\,E=\frac{S\,S\,t\,o\,t\,a\,l-M\,S\,R*\left(n-1\right)-M\,S\,C*\left(k-1\right)}{\left(n-1\right)*\left(k-1\right)}$$

$$\begin{array}{c} S\,S\,t\,o\,t\,a\,l=v\,a\,r\,{\left(x\,\left(:\right)\right)}^{*}\,\left(n^{*}\,k-1\right)\\ M\,S\,R=v\,a\,r\,{\left(m\,e\,a\,n\,\left(x,2\right)\right)}^{*}\,k\\ M\,S\,C=v\,a\,r\,{\left(m\,e\,a\,n\,\left(x,1\right)\right)}^{*}\,n \end{array}$$

where x is the data matrix.

ICC scores typically range between −1 and 1. However, in this manuscript the values have been scaled to a range of −100 and 100, resulting in the following categorization: poor (ICC < 21), fair (20 < ICC < 41), moderate (40 < ICC < 61), substantial (60 < ICC < 81), and almost perfect (ICC > 80) (Landis and Koch, 1977).

In order to formally compare ICC scores across modalities, *F*-tests were run for each LEiDA metric, testing the null hypothesis that the ICC score of a given modality was equal to that of another modality, in line with previous work by McGraw and Wong (1996). FDR correction (Benjamini and Hochberg, 1995) was used for adjusting for all four LEiDA states and all six contrasts (i.e., SB-ASSET2 compared to MB4-ARC1, MB4-ARC2, and MB6-ARC1; MB4-ARC1 compared to MB4-ARC2 and MB6-ARC1; and MB4-ARC2 compared to MB6-ARC1.

## Results

### Motion characteristics of the sample

In line with previous studies (Hallquist et al., 2013; Muschelli et al., 2014), there was a significant reduction in the median correlation between FD and DVARS after denoising compared to before within all four rs-fMRI modalities, FDR-corrected. Plots of the distributions across subjects before and after denoising are provided in Supplementary Figure 2. With regards to the Coefficients of Determination, there was also a significant reduction in how much variability in DVARS is explained by FD after denoising compared to before, for each of the four rs-fMRI modalities, FDR-corrected (Supplementary Figure 3).

### Dynamic analyses

The Dunn score revealed an optimal solution with *k* = 6 clusters for single band and *k* = 5 clusters for all three MB modalities.

### Comparing clusters across all four resting-state functional magnetic resonance imaging modalities and in relation to reference functional networks

**FIGURE 2:**
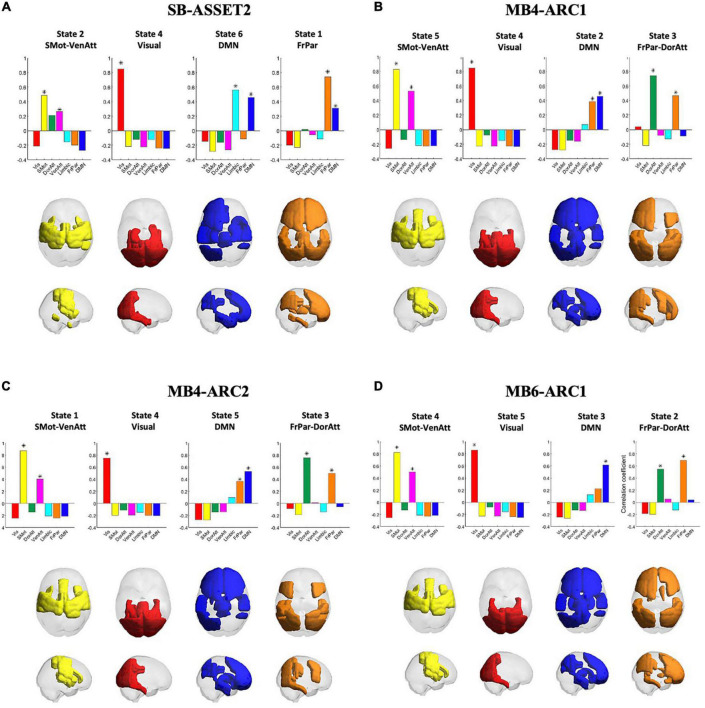
Consistency of cluster centroids detected across modalities. The four panels represent the patterns detected in each fMRI acquisition modality, namely SB-ASSET2 **(A)**, MB4-ARC1 **(B)**, MB4-ARC2 **(C)**, and MB6-ARC1 **(D)**. Bar graphs at the top of each panel report the Pearson’s correlation coefficients between the patterns representative of each state and seven functional networks used as reference defined by Yeo et al. (2011). Each state is represented by rendering the subset of brain areas with positive values in the leading eigenvector of that state on a transparent cortex displayed in axial and saggital views. Vis, visual network; SMot, somato-motor network; DorAtt, dorsal attention network; VenAtt, ventral attention network; FrPar, frontoparietal network. *Represents a significant correlation between a given state and a Yeo network.

Pearson’s correlation coefficients between clusters, or states, across all four modalities are provided in the Supplementary Figure 4. A significant correlation (*p* < 0.01/k) was observed across all four fMRI sequences only for the somato-motor and ventral attention networks, the visual network, the DMN and the frontoparietal and dorsal attention networks, and therefore only these networks were taken forward to the ICC analysis. The rendering of each of these states on the cortex across all four modalities, in addition to Pearson’s correlation coefficients between each of these states and each of the seven Yeo networks, is presented in Figure 2. Colored areas represent the brain regions whose phase positively projects onto the leading eigenvector of that state. Because the k-means clustering was run separately for each of the four modalities, the networks were ordered differently in the k-means output across modalities. This explains why the number attributed to each state varies across modalities. In the rest of the paper, we will refer to each state by the Yeo reference network (Yeo et al., 2011) they most correlate with in order to make figures and tables easier to interpret.

For each of the four states taken forward to the ICC analyses, there were a few differences across the four modalities in terms of which ROIs positively contributed to each state. First, for the somato-motor and ventral attention network, the left posterior temporal gyrus and the frontal operculum cortex positively contributed only to all three MB modalities; the left anterior supramarginal gyrus positively contributed only to SB-ASSET2, MB4-ARC1, and MB6ARC1; and the anterior temporal fusiform cortex positively contributed only to SB-ASSET2.

With regards to the visual network, the superior parietal lobule positively contributed only to MB6-ARC1; and the posterior parahippocampul gyrus positively contributed only to SB-ASSET2, MB4-ARC1, and MB4-ARC2.

For the DMN, the right frontal pole, the right superior frontal gyrus, the inferior frontal gyrus triangularis, the frontal operculum cortex, the thalamus and the caudate positively contributed positively contributed only to all three MB modalities, while the temporal pole, the parahippocampal gyrus, the temporal fusiform cortex, the hippocampus and the amygdala positively contributed only to SB-ASSET2.

Finally, for the frontoparietal and dorsal attention networks, the superior frontal gyrus, the left anterior inferior temporal gyrus and the paracingulate gyrus positively contributed only to SB-ASSET2; the frontal pole, the occipital fusiform gyrus and the caudata positively contributed only to SB-ASSET2 and MB6-ARC1; and the superior parietal lobule, the inferior lateral occipital cortex, the posterior temporal fusiform cortex and the temporooccipital part of the inferior temporal gyrus positively contributed only to MB4-ARC1 and MB4-ARC2.

### Intraclass correlation coefficient analysis

**FIGURE 3:**
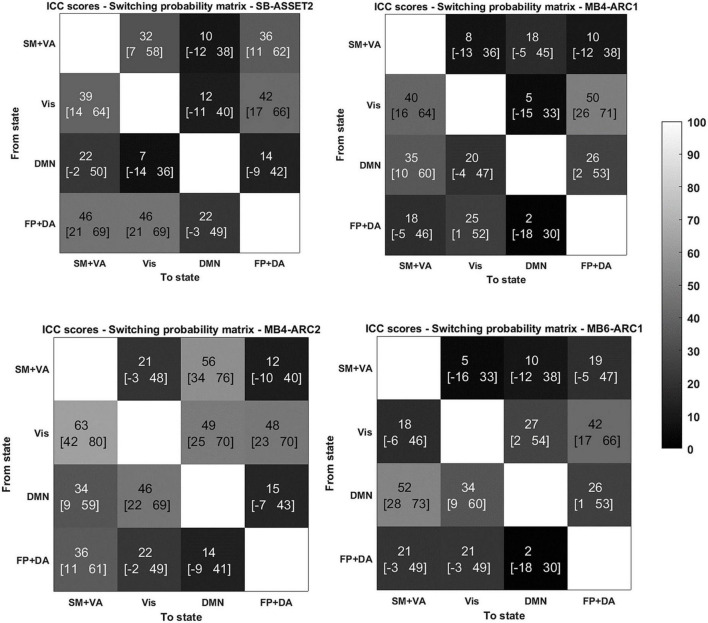
Reliability of switching probabilities between states for the 4 modalities. ICC scores (lower bound of the 95% confidence interval- upper bound of the 95% confidence interval) for the switching probability measure from and to each of the states for SB-ASSET2, MB4-ARC1, MB4- ARC2, and MB6-ARC1; color-coded based on Landis and Koch (1977)’s ICC categorization: poor in blue, fair in green, moderate in orange, substantial in red, and almost perfect in brown. SM, somato-motor network; VA, ventral attention network; FP, frontoparietal network; DA, dorsal attention network; Vis, visual network.

In order to identify the test-retest reliability of all three LEiDA metrics (i.e., probability of occurrence, lifetime, and switching probability), we calculated the ICC scores of each measure across all three runs for each network and each modality. Full details of the ICC scores and associated confidence intervals are provided in Table 2 and Figure 3. Overall, ICC scores ranged between “fair” and “substantial” for the probability of occurrence and between “poor” and “substantial” for the lifetime and switching probability measures across all four modalities.

**TABLE 2:** Reliability of probabilities and lifetimes across three time points for the four modalities.

Yeo networks	SMot and VenAtt	Visual	DMN	FrPar and DorAtt
	SB-ASSET			
ICC_probability (LB−UB)	39 (14–64)	52 (28–73)	24 (0–51)	51 (27–73)
ICC lifetime (LB−UB)	25 (1–53)	33 (7–59)	33 (8–59)	51 (27–72)
	MB4-ARC1			
ICC probability (LB−UB)	40 (16–64)	59 (37–76)	57 (34–76)	40 (15–63)
ICC lifetime (LB−UB)	48 (24–69)	38 (14–62)	59 (37–77)	42 (18–65)
	MB4-ARC2			
ICC probability (LB−UB)	54 (30–73)	77 (60–87)	50 (25–70)	52 (28–72)
ICC lifetime (LB−UB)	49 (26–69)	65 (45–81)	41 (16–64)	49 (26–70)
	MB6-ARC1			
ICC probability (LB−UB)	38 (13–62)	60 (37–77)	57 (34–76)	32 (7–57)
ICC lifetime (LB−UB)	42 (17–65)	56 (33–75)	47 (23–69)	47 (23–69)

ICC scores (lower bound of the 95% confidence interval—upper bound of the 95% confidence interval) for the probability of occurrence and the lifetime for each of the four modalities across the three visits for single band (SB-ASSET2) and Multiband MRI acquisitions (MB4-ARC1, MB4-ARC2, and MB6-ARC1); color-coded based on Landis and Koch (1977)’s ICC categorization: poor in blue, fair in green, moderate in orange, substantial in red, and almost perfect in brown; FDR-corrected.

More specifically, for the probability of occurrence metric, 6 out of 12 ICC values fell into the moderate range (40 < ICC < 61) and 2 out 12 fell into the substantial range (60 < ICC < 81) for MB, while 2 out of 4 values fell into the moderate range for single band. For the lifetime, 10 out 12 values fell into the moderate range and 1 score fell into the substantial range for MB, while only 1 out of 4 values fell into the moderate range for single band. For the switching probability, 7 out 36 values fell into the moderate range and 1 fell into the substantial range for MB, and 3 out of 12 values fell into the moderate range for single band.

**FIGURE 4:**
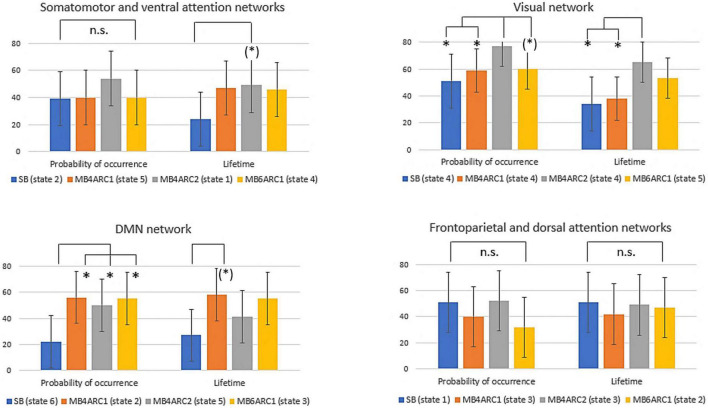
Formal comparison of the ICC scores and associated confidence intervals for the probability of occurrence and lifetime measures across all four rs-fMRI modalities. n.s., non-significant; (*), significant *F*-test, FDR-uncorrected (did not survive FDR correction); *, significant *F*-test, FDR- corrected (survived FDR correction).

Formal comparison of the ICC scores across modalities revealed different patterns of reliability across networks and LEiDA measures (see Table 2 and Figure 4).

With regards to the somato-motor and ventral attention networks, there was no significant difference across modalities for the probability of occurrence. For the lifetime, the *F*-test revealed significantly higher reliability scores MB4-ARC2 (state 1) compared to SB-ASSET2 (state 2), however, it did not survive pFDR correction [*F*(23, 46) = 3.66; *p* = 0.02, pFDR > 0.05]. The ICC score for switching probability from these networks to the DMN was significantly more reliable for MB4-ARC2 compared to all other three modalities (*F* = 4.8; *p* = 0.00006 with SB-ASSET2; *F* = 2.94; *p* = 0.0007 with MB4-ARC1; *F* = 3.66; *p* = 0.00006 with MB6-ARC1). No significant difference across modalities was found for the transition from these networks to the visual networks and the frontoparietal and dorsal attention networks.

Additionally, the visual network yielded significantly higher ICC scores with MB4-ARC2 (state 4) compared to SB-ASSET2 for the probability of occurrence measure [*F*(23, 46) = 2.53, *p* = 0.002, pFDR < 0.05] and MB4-ARC1 [*F*(23, 46) = 2.02, *p* = 0.01, pFDR < 0.05]. The F-test also revealed significant differences between the ICC scores for MB4-ARC2 and MB6-ARC1, however, it did not survive FDR correction [*F*(23, 46) = 1.95, *p* = 0.02, pFDR > 0.05]. With regards to the lifetime measure, the visual network showed significantly increased reliability with MB4-ARC2 compared to SB-ASSET2 [state 4, *F*(23, 46) = 2.70, *p* = 0.001, pFDR < 0.05] and MB4-ARC1 [state 4, *F*(23, 46) = 2.35, *p* = 0.004, pFDR < 0.05] only. The switching probability from the visual network to the DMN was significantly more reliable with MB4-ARC2 compared to SB-ASSET2 [*F*(23, 46) = 2.72; *p* = 0.001, pFDR < 0.05] and MB4-ARC1 [*F*(23, 46) = 3.32; *p* = 0.0001, pFDR < 0.05]. Furthermore, the switching probability from this network to the somato-motor and ventral attention networks was significantly more reliable with MB4-ARC2 compared to MB6-ARC1 [*F*(23, 46) = 3.70, *p* = 0.00005, pFDR < 0.05] and also significantly more reliable compared to SB-ASSET2 [*F*(23, 46) = 2.1, *p* = 0.01] and MB4-ARC1 [*F*(23, 46) = 2.04, *p* = 0.01] but did not survive FDR correction for these latter two modalities (pFDR > 0.05).

Furthermore, the probability of occurrence of the DMN was significantly more reliable for SB-ASSET2 compared to MB4-ARC1 [*F*(23, 46) = 2.55, *p* = 0.002, pFDR < 0.05], MB4-ARC2 [*F*(23, 46) = 2.01, *p* = 0.01, pFDR < 0.05] and MB6-ARC1 [*F*(23, 46) = 2.46, *p* = 0.003, pFDR < 0.05]. In contrast, only MB4-ARC1 exhibited significantly higher ICC scores compared to SB-ASSET2 for the lifetime, however, it did not survive FDR correction [*F*(23, 46) = 2.13, *p* = 0.01, pFDR > 0.05]. Additionally, the switching probability from the DMN to the somato-motor and ventral attention networks was significantly more reliable with MB6-ARC1 compared to SB-ASSET2 [F(23, 46) = 2.30; *p* = 0.007, pFDR < 0.05]. In contrast, the switching probability from the DMN to the visual network was significantly more reliable with MB4-ARC2 compared to SB-ASSET2 [*F*(23, 46) = 2.91; *p* = 0.0007, pFDR < 0.05] and MB4-ARC1 [*F*(23, 46) = 2.04; *p* = 0.01] but did not survive FDR correction for MB4-ARC1 (pFDR > 0.05).

Finally, with regards to the frontoparietal and dorsal attention networks, there were no differences across all four modalities for the probability of occurrence, the lifetime or the switching probability from and to these states.

## Discussion

To our knowledge, this study was the first to compare the test-retest reliability of all three LEiDA measures (i.e., the probability of occurrence, the lifetime, and the switching probability) across a conventional single-band fMRI and three different MB acquisitions, with and without in-plane acceleration, across three visits. Previous research has suggested that an acceleration factor of 8 yields satisfactory ICC scores across all three measures (Vohryzek et al., 2020). However, no study to date had explored which combination of MB and in-plane acceleration factors give the best results in comparison with single band sequences.

The main finding of this study is that, for all three LEiDA measures, ICC scores were higher for all three MB modalities compared to single band. These findings concur with our first hypothesis that ICC scores would be significantly higher across all three MB modalities compared to single band and may reflect MB sequences’ key contribution to faster sampling rate leading to the acquisition of a greater number of timepoints and increased detection of subtle changes in dynamic spatio-temporal patterns of brain activity. Improved reliability with MB was observed for the visual network, the DMN and the somato-motor and ventral attention networks. It is worth noting that all these networks are cortical networks. These findings therefore accord with previous studies emphasizing the key role of MB sequences in improving test-retest reliability of activity in cortical structures (Cahart et al., 2022). Additionally, in the present study, the limbic network, which includes subcortical structures such as the amygdala and the nucleus accumbens, correlated with single band’s state 6 but did not correlate with any of the MB states and therefore was not included in our subsequent ICC analyses. It is important to mention that, even when we imposed six states (e.g., *k* = 6) during state selection for the MB sequences, the limbic network still represented the only network that did not correlate with any of the states. This suggests that MB sequences reduce LEiDA’s ability to detect the limbic network, which is consistent with a large body of evidence showing that subcortical structures are more prone to reduced signal to noise ratio in the context of MB compared to single band (Risk et al., 2021; Cahart et al., 2022). This could also explain why the limbic network was not successfully detected in previous MB studies using LEiDA, unless the number of states (k) was strictly higher than 8 (Vohryzek et al., 2020).

Furthermore, for the probability of occurrence and lifetime measures, it is important to mention that most MB ICC scores exceeded 40 (i.e., 0.40) and fell into the moderate range, which is in line with previous findings (Vohryzek et al., 2020), except for MB4-ARC2 where values reached the substantial range (60 < ICC < 81) for some of the states. Overall, these results outperform ICC values reportedly obtained with other dynamic FC methods such as sliding windows (Choe et al., 2017). Indeed, approximately 75% of ICC values derived from the variance of ROI-to-ROI connectivity across short sliding windows of fixed length of time have previously been observed to fall below 40 when the data was acquired with a standard single band sequence and up to 100% of the ICC values fell under 40 for sequences with an acceleration factor of 8, depending on the window length (Choe et al., 2017). These results support the key role of LEiDA as an alternative to other dynamic approaches when it comes to reliably detecting brain dynamics.

Additionally, for the probability of occurrence and lifetime, the pattern of reliability values across rs-fMRI modalities varied by networks. More specifically, with regards to the lifetime of the DMN, scores were significantly higher with MB4-ARC1 compared to the single band modality, which fits in with our second hypothesis, even though the difference did not survive FDR correction. In contrast, and contrary to our expectations, MB4-ARC2 yielded higher scores compared to SB-ASSET2 and MB4-ARC1 across both metrics for the visual network. In the context of the switching probability measure, scores also tended to be significantly higher with MB4-ARC2 compared to the other sequences for transitions across most states except for transitions from and to the frontoparietal and dorsal attention networks where no significant differences were observed across modalities. These findings suggest that the combination of increased MB factor (i.e., MB4) with in-plane acceleration (i.e., ARC2) may not only capture time-varying patterns of transitions more accurately through faster sampling compared to SB-ASSET2 but also limit susceptibility distortions, thus improving reliability scores.

Taken together, the present results demonstrate that MB acceleration does improve ICC scores across all LEiDA measures and most cortical networks. In particular, MB acceleration might benefit studies exploring task-negative networks (i.e., DMN) which have been shown to be implicated in psychiatric disorders such as major depressive disorder (Wise et al., 2017), post-traumatic stress disorder (Fu et al., 2019), and schizophrenia (Weber et al., 2020). MB sequences might also improve reliability scores for other networks such as the somato-motor and ventral attention networks which are known to be involved in reorienting one’s attention toward salient stimuli and have previously been implicated in Alzheimer’s Disease (Gu et al., 2020), eating disorders (Spalatro et al., 2019), trait impulsivity (Herman et al., 2020), and adolescent depression (Liu et al., 2019).

Another key finding of this study is that the probability of occurrence and lifetime measures showed reliability scores ranging between fair and substantial across all four modalities while the switching probability exhibited a more heterogeneous profile of ICC scores ranging between poor and substantial across all four modalities. It is worth highlighting that a few factors might influence the ability of dynamic fMRI measures to reliability detect meaningful patterns of brain activity and disease biomarkers. Here, substantial ICC scores for the probability of occurrence and lifetime of some of the states might not only indicate that these LEiDA metrics reliably capture dynamic FC patterns of brain activity over time but may also suggest that the participants’ psychological and physiological states were fairly stable across the three sessions. In contrast, poor ICC scores observed for the switching probability measure suggest that participants transitioned between states in a highly different manner from one scan to the next, thus increasing within-subject variability across visits. These findings indicate that future studies using this LEiDA metric may interpret findings with caution.

### Limitations

One limitation of our study is that the cerebellar ROIs were excluded from our analyses due to their absence in the Yeo parcellations (Yeo et al., 2011). Future studies could consider using different parcellations that include the cerebellar networks, as cerebellar dysfunction is known to play a key role in some neurodevelopmental and psychiatric disorders (Phillips et al., 2015).

Additionally, for this study, aCompcor was carried out before band-pass filtering, rather than simultaneously, which we are aware may be accompanied by higher nuisance-related variability in the resting-state frequencies of interest (Hallquist et al., 2013). Recent studies have shown that neither of these approaches (i.e., regression followed by bandpass filtering or simultaneous execution of both processes) have been associated with a poor attenuation of nuisance signals; and connectivity estimates for both models have been shown to be very similar, with no evidence of global signal changes of large magnitude that would appear to be in temporal synchrony with head motion (Hallquist et al., 2013). However, it has been suggested that carrying out high-pass filtering after motion regression may reintroduce artifacts into the data, and performing a simultaneous regression on all nuisance covariates may limit these issues (Lindquist et al., 2019). Considering these findings, future studies should consider using the “Simult” option implemented in CONN.

Furthermore, several studies have shown that head motion and physiological artifacts such as cardiac and breathing variations tend to be strongly linked to the variance in the global fMRI signal (Power et al., 2017) and correlate with patterns in time-varying FC (Xifra-Porxas et al., 2021), thus affecting FC measures. Physiological corrections have been shown to decrease ICC scores, suggesting that subject specificity in FC measures is partly due to the presence of subject-specific motion and physiological artifacts which complicates somewhat the assessment of data quality based on test-retest reliability measures. For this study, quality control metrics revealed a significant reduction in the median correlations between FD and DVARS after denoising compared to before for most of the runs within all four rs-fMRI modalities using a standard aCompCor approach. However, despite reducing the median FD-DVARS correlations more effectively than when regressing out the mean signals, the standard aCompCor approach has been shown to be outperformed by pre-processing strategies such as aCompCor50 where a higher number of principal components are included in the regression (Muschelli et al., 2014). Future studies may consider using more aggressive pipelines to further remove physiological noise and increase the validity of FC measures.

Finally, participants in this study were healthy and neurotypical and therefore we can’t generalize our findings to clinical populations. It could be argued that ICC scores for some of the LEiDA measures could be different, and potentially higher, in clinical populations that tend to exhibit repetitive thoughts, ritualistic behaviors or inflexible autonomic responding such as individuals with depression, autism or obsessive-compulsive behaviors (Borkovec, 1994; Thayer et al., 1996; Kashdan, 2010). Future studies would benefit from investigating the contribution of specific psychological and physiological processes in modulating the test-retest reliability of each of the LEiDA metrics in the context of psychiatric disorders compared to healthy controls.

## Conclusion

In conclusion, this study was the first, to our knowledge, to compare ICC scores across four different rs-fMRI modalities and across three visits in the context of the LEiDA measures. We found strong evidence that MB modalities yield significantly higher reliability scores compared to single band across all three functional measures for several cortical networks. Finally, our findings also revealed that MB acquisition hinders the detection of subcortical networks in the context of LEiDA, which suggests that studies with a specific focus on subcortical structures might consider choosing single band acquisition over MB sequences. Future research exploring other combinations of acceleration and different brain parcellations would further shed light on the different factors at play in the context of the reliability of all three LEiDA measures.

## Data availability statement

The raw data supporting the conclusions of this article will be made available by the authors, without undue reservation.

## Ethics statement

The studies involving human participants were reviewed and approved by the King’s College London Research Ethics Committee (HR-17/18-5720). The patients/participants provided their written informed consent to participate in this study.

## Author contributions

SW, FD’A, MT, JS, and SE contributed to the conception and design of the study. FZ contributed to the design of the study. M-SC collected the data, carried out the statistical analyses and wrote the first draft of the manuscript. OO’D and JC contributed to the statistical analyses and interpretation of the data. VG contributed to the interpretation of the data. All authors contributed to the manuscript revision and approved the submitted version.

## Footnotes

1
http://www.nitrc.org/projects/artifact_detect
