# Supplementary figures and images for: Test-retest reliability of time-varying patterns of brain activity across single band and multiband resting-state functional magnetic resonance imaging in healthy older adults
Source: Front Hum Neurosci. 2022 Nov 10;16:980280. doi: 10.3389/fnhum.2022.980280 (PMC9685802; doi:10.3389/fnhum.2022.980280)

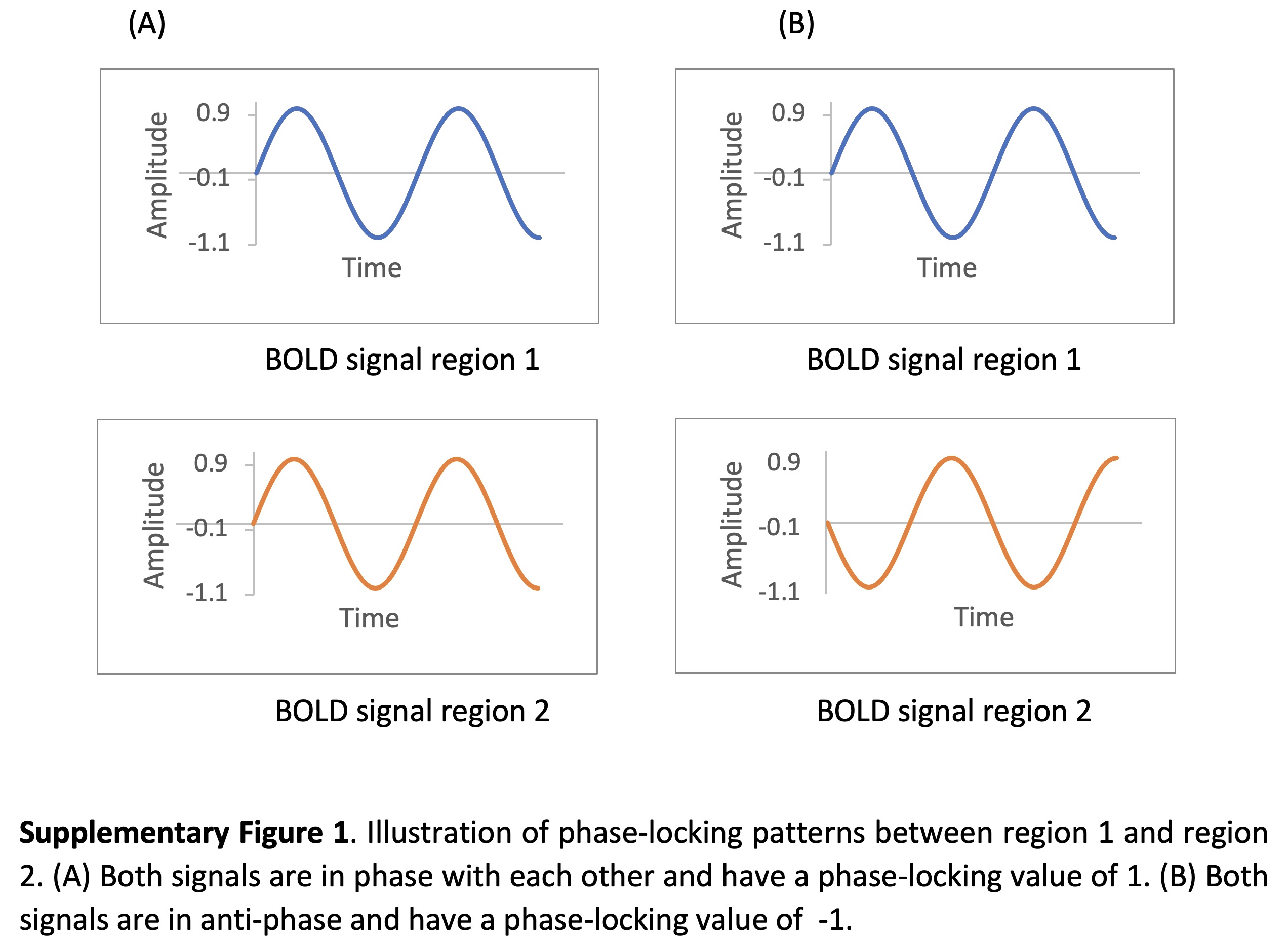

Supplement: Supplementary file 1 [file Image_1.JPEG]

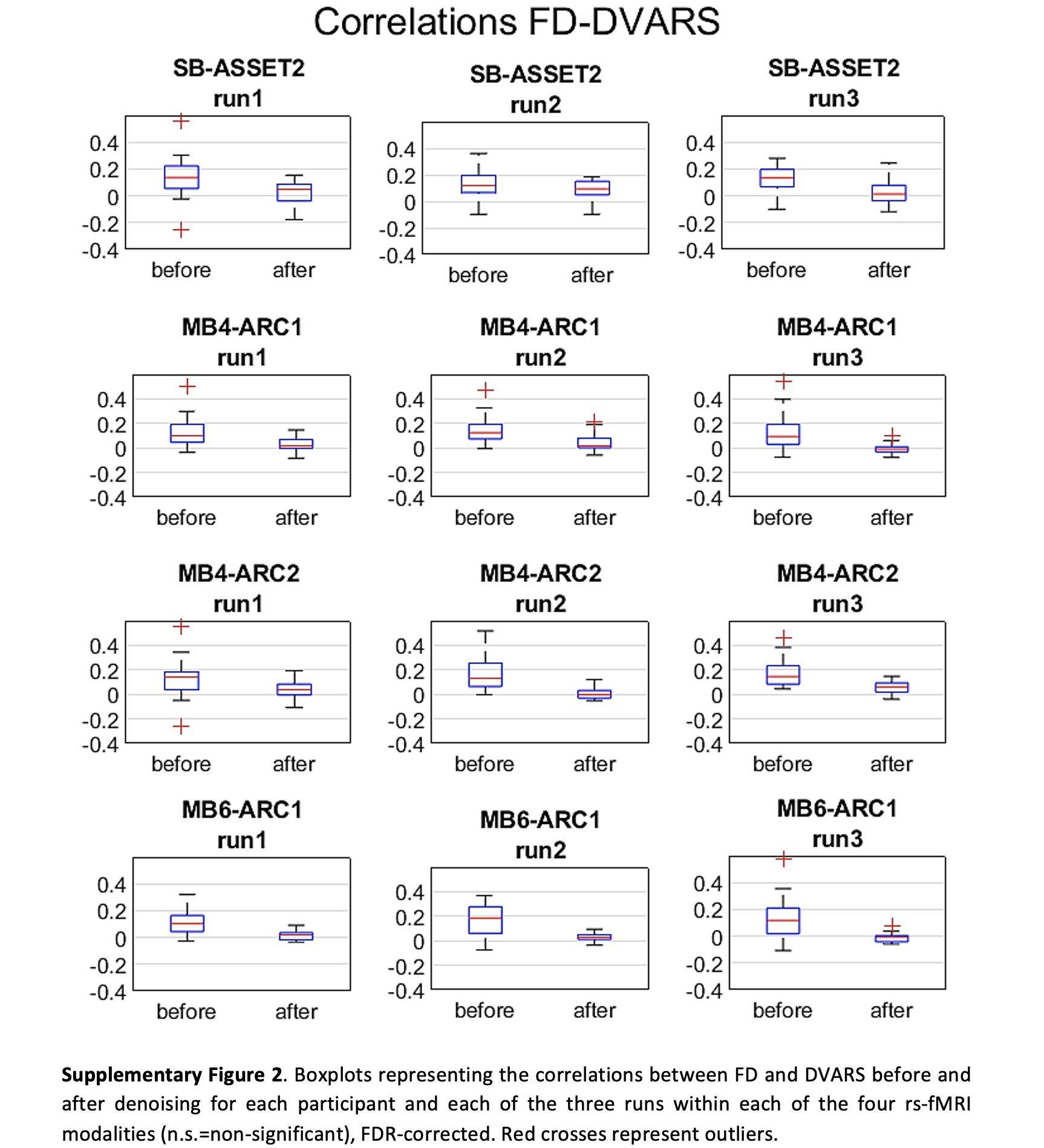

Supplement: Supplementary file 2 [file Image_2.JPEG]

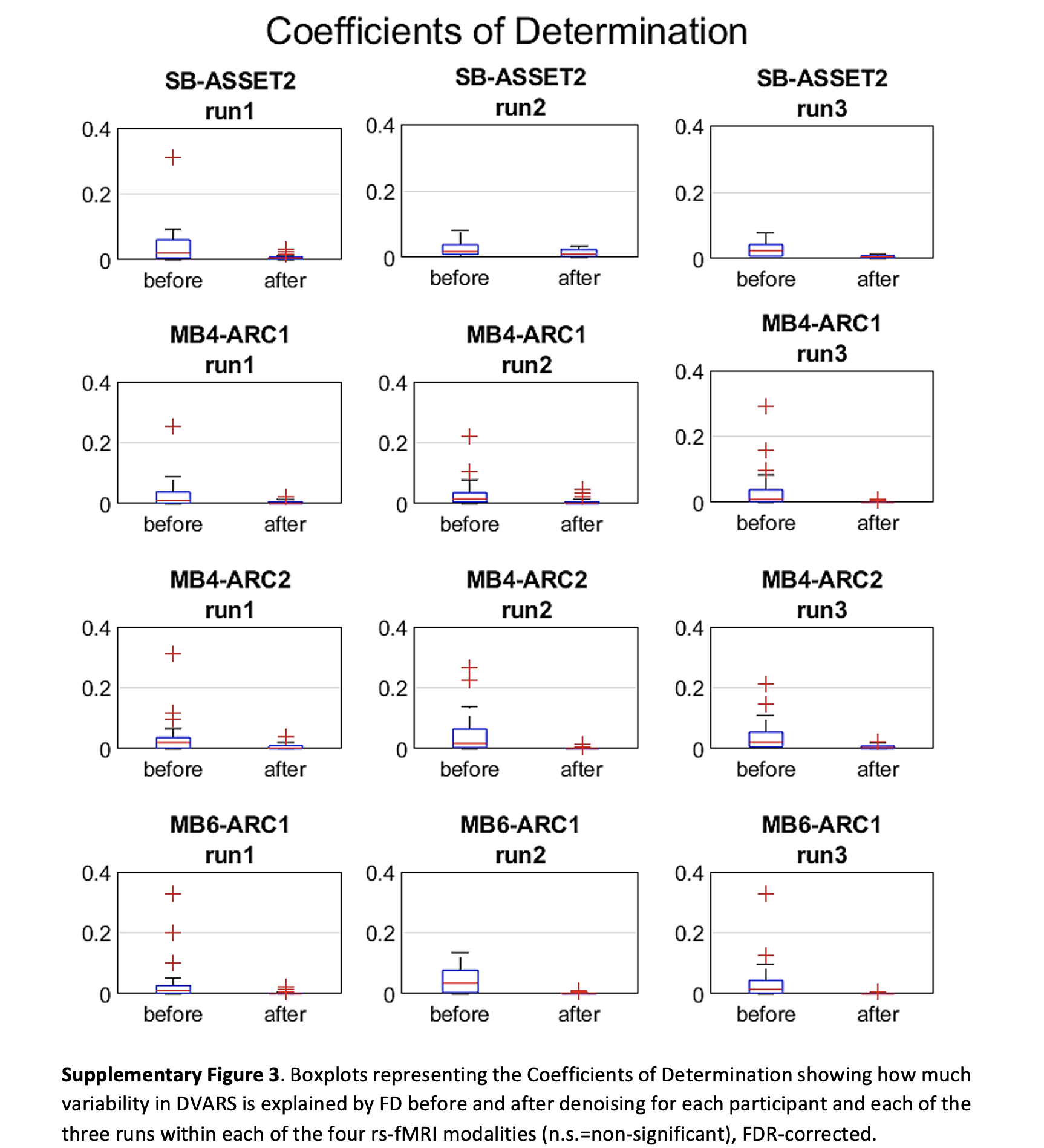

Supplement: Supplementary file 3 [file Image_3.JPEG]

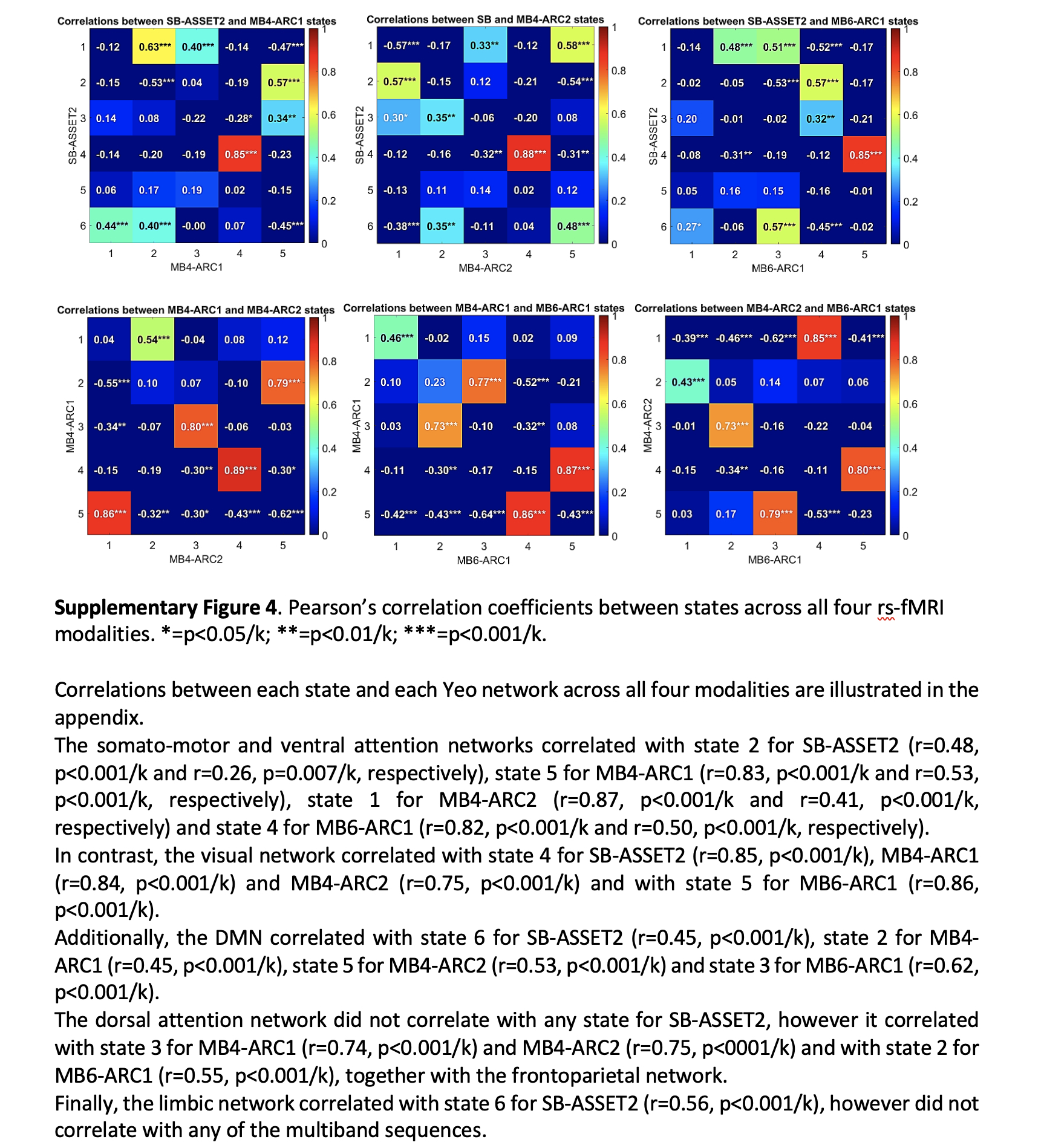

Supplement: Supplementary file 4 [file Image_4.JPEG]
